# A novel and effective method for validation and measurement of output factors for Leksell Gamma Knife® Icon™ using TRS 483 protocol

**DOI:** 10.1002/acm2.13011

**Published:** 2020-09-06

**Authors:** Swapna Lilly Cyriac, Jian Liu, Emel Calugaru, Jenghwa Chang

**Affiliations:** ^1^ Department of Radiation Oncology KIMS Cancer Care and Research Center Pvt Ltd Thiruvananthapuram Kerala India; ^2^ Department of Radiation Medicine Northwell Health Lake Success NY USA; ^3^ Department of Radiation Medicine Donald and Barbara Zucker School of Medicine at Hofstra/Northwell Lake Success NY USA; ^4^ Department of Physics and Astronomy Hofstra University Hempstead NY USA

**Keywords:** diode detectors, effective point of measurement, relative output factors, small field dosimetry, TRS‐483

## Abstract

The objective of this work was to identify the exact location of the effective point of measurement (EPM) of four different active detectors to compare the relative collimator output factors (ROF) of Leksell Gamma Knife (LGK) according to IAEA TRS‐483 recommendations. ROF was measured at the center of the spherical LGK‐Solid Water (LGK‐SW) Phantom for three (4‐, 8‐, and 16‐mm in diameter) collimators using four (PTW‐TN60008, PTW‐TN60016, PTW‐TN60017, and PTW‐60019 diode/diamond) detectors. Since diode detectors have a much smaller sensitive volume than the PTW‐31010 ion chamber used for reference dosimetry, its EPM might not be at the center of the phantom, or (100, 100, 100) of the Leksell Coordinate System, particularly in the *z‐*direction. Hence for each diode detector, a CBCT image was acquired after it was inserted into the phantom, from which the z‐Leksell coordinate of EPM was determined. Relative collimator output factors was then measured by focusing GK beams on the determined EPM of each diode. Measured ROFs were compared with the vendor‐provided values in GK treatment planning system. For validation, a plan was generated to measure the output of 4‐mm collimator for PTW‐TN60017 at various couch locations along the z‐axis. For PTW‐TN60008, the percentage variations were 0.6 ± 0.4%, and −0.8 ± 0.2% for 4 and 8‐mm collimators, respectively. For PTW‐TN60016, the percentage variations were 0.8 ± 0.0%, and 0.2 ± 0.1%, respectively. The percentage variations were −3.3 ± 0.0% and −0.9 ± 0.1%, respectively, for PTW‐TN60017, and −0.5 ± 0.0% and −0.8 ± 0.2%, respectively, for PTW‐TN60019. Center of the measured profile for PTW‐TN60017 was only 0.1 mm different from that identified using the CBCT. In conclusion, we have developed a simple and effective method to determine the EPMs of diode detectors when inserted into the existing LGK‐SW phantom. With the acquired positional information and using TRS‐483 protocol, good agreements were obtained between the measured ROFs and manufacturer recommended values.

## 1 INTRODUCTION

Small photon fields are widely used in stereotactic radiosurgery (SRS) and radiotherapy (SRT). Dose measurements of small field sizes are challenging, due to many factors such as source occlusion, detectors size limitation, and lack of lateral electronic equilibrium.^1^, ^2^ Hence a comprehensive quality assurance (QA) program for SRS and SRT is critical to ensure the correct dose being delivered to the target, given the very small target volumes and rapid dose fall‐off associated with this stereotactic delivery. The new codes of practice (COP), Technical Report Series No‐ 483 (TRS‐483) jointly developed by International Atomic Energy Agency (IAEA) and American Association of Physicists in Medicine (AAPM),^3^ provides guidance for measurements of field output factors and lateral beam profiles at the measurement depth for such small fields.

In small fields, the precise and accurate determination of the effective relative output factor (ROF) of a Leksell Gamma Knife (LGK) system (Elekta AB, Stockholm, Sweden) is a very challenging task due to the extremely narrow beams. Several studies have reported that ROF of small fields measured with different active and passive detectors can differ up to 5% for 4mm collimator, and most of them have reported for the earlier models of LGK.^4^, ^5^ Araki et al performed measurements for ROF using radiophoto luminescent glass rod dosimeters and p‐type silicon diode detector for the model B Leksell Gamma Knife and showed good agreement with manufacturer’s recommended values.^6^ Kurjewicz et al used metal‐oxide‐semiconductor field effect transistors (MOSFETs) to measure ROF with results in agreement with manufacturer recommended values.^7^ ^.^Whereas Novotny Jr. et al used three types of film to measure the ROF of 4 and 8 mm collimator of the LGK Perfexion and have obtained output factors with a maximum deviation of −4.5% with the Monte Carlo calculated value for the 4 mm collimator.^8^

Hrsak et al studied the angular dependence and end effect time for p‐type silicon detectors and PinPoint ionization chamber with LGK.^9^ The corrected ROFs for 14 and 8 mm collimator were in a very good agreement with the recommended values. However, for the 4‐mm collimator, the diodes showed over response of maximum 2.8% and for the pinpoint chamber ROF was 25% lower than the vendor value. Thus, the size and type of detectors used for the ROF measurements are critical to the accuracy of measurement, and detectors with high spatial resolution, high precision, and good tissue equivalence are required.^10^

In addition to detector response, identifying the EPM of each detector is necessary as the whole profile is a small field and the EPM is usually in the penumbra region. When the phantom is docked in the Gamma Knife for irradiation, the center of the phantom coincides with the focal point of the LGK unit, or (100, 100, 100) of the Leksell Coordinate System (LCS) in units of millimeters. However, when a detector is inserted at this focal point, its reference point of measurement may not coincide with the focal point.

In this paper, we investigated how to determine the EPM of different diode detectors for accurate measurements of ROFs for LGK Icon™ unit. In addition, ROFs were measured using four different active detectors according to the recommendations of IAEA TRS‐483 report. The results were compared with the vendor‐recommended values in the Gamma Knife planning (GKP) system.

## 2 METHODS

### 2.A Leksell Gamma Knife® icon

The Leksell Gamma Knife (LGK) Icon (Elekta AB, Stockholm, Sweden) is the most recent version of Leksell Gamma Knife units for brain radiosurgery. It mainly differs from its predecessor LGK Perfexion unit because of the addition of a KV‐ CBCT and an intra‐fraction High‐Definition Motion Management (HDMM) system based on infra‐red (IR) light.

The on‐board CBCT system uses an x‐ray source with energies ranging from 70 to 120 kVp and a spot size of 0.6 mm. The imaging panel is made of an array (780 × 720) of amorphous Si detectors with a pixel size of 0.368 × 0.368 mm^2^. The reconstructed CBCT volume is 224 × 224 × 224 mm^3^ with a voxel size of 0.5 × 0.5 × 0.5 mm^3^. The integrated CBCT system is calibrated to use the Leksell Coordinate System providing stereotactic reference images for the treatment and for treatment planning.^11^

The HDMM system consists of an IR camera, an IR reference tool and a patient marker. It is not used for initial setup of the patient but for monitoring the patient movements during the treatment. After the patient is aligned using the CBCT, the IR camera tracks the patient maker at a frequency of 20 Hz and measure its movement relative to the reference coordinate system defined by the IR reference tool. Treatment will be held off if the patient motion exceeds the preselected threshold, and resumed again when the patient returns to the initial position.

Both CBCT and HDMM systems have been introduced to provide LGK accuracy for fractionated stereotactic radio surgery (SRS) treatments that involve the replacement of the invasive coordinate frame with the use of thermoplastic masks. Different types of thermoplastic masks are commercially available from numerous vendors. They are specially designed for SRS/SRT, which are stiffer, Kevlar reinforced, and have less shrinkage.

### 2.B Gamma knife solid water (SW) phantom

**Fig. 1:**
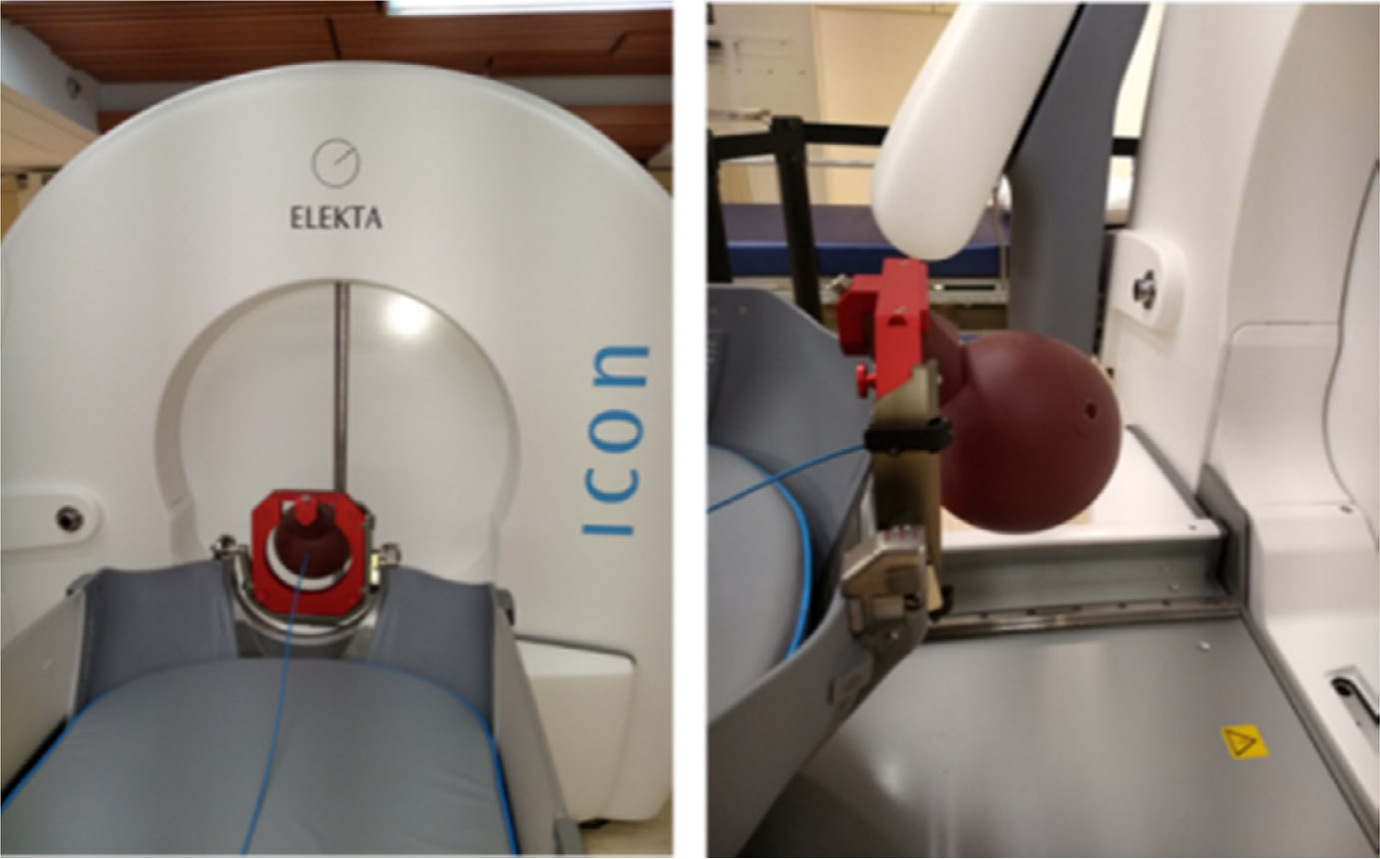
Setup for the measurement of relative output factors in the Gamma Knife Icon at the Centre of Advanced Medicine of Northwell Health. The diode detectors were inserted into the Leksell Solid Water (SW) phantom to measure the ionization at the center of the detector for the 16 mm, 8‐mm and 4‐mm shots.

Relative output factor (ROF) measurements were performed on the LGK Icon unit at the Center for Advanced Medicine (CFAM) of Northwell Health (New York, US) using the spherical Leksell Gamma Knife Solid Water (SW) Dosimetry Phantom provided by the manufacturer. When the phantom is docked to the machine, the Leksell coordinates of its center are (100, 100, 100) in millimeter. Figure 1 illustrates how the detector was inserted into the SW phantom and stabilized with tape for output measurements. The LGK SW phantom is designed for output measurement using PTW‐31010 Semiflex ionization Chamber (sensitive volume: 0.125 cc, Dimension: radius 2.75 mm, length 6.5 mm). That is, when inserted into the phantom, the center of this ionization chamber, or effective point of measurement (EPM) is located at the phantom center with Leksell coordinates of (100,100,100). For routine output measurement, a treatment plan is executed to move the couch inside the GK unit to a location so that the planned location, that is, (100,100,100), coincides with the radiological focus of the machine known as the unit center point (UCP). The irradiation time can be controlled by the treatment plan or using the dose integration function of the electrometer, in which the user can specify a time (e.g., 1 min) for charge collection.

### 2.C Diode detectors for ROF measurements

**Fig. 2:**
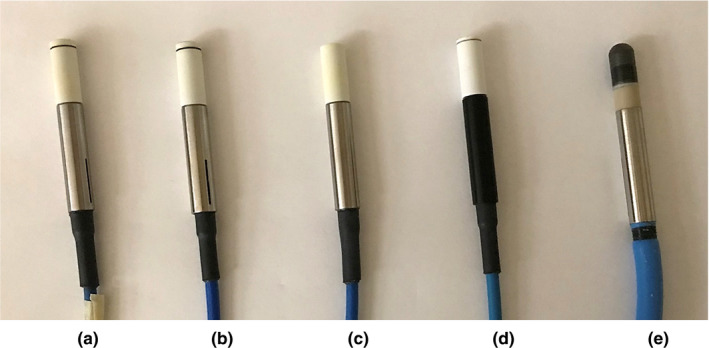
Illustrations of the four detectors used in this study: (a) PTW‐TN60008 diode detector, (b) PTW‐TN60016 diode detector, (b) PTW‐TN60017 diode detector, and (d) PTW‐60019 diamond detector. These four detectors have the‐ same outer geometry (diameter 7 mm) but different internal structures. Also shown in (e) is the PTW‐31010 ionization chamber used for the reference dosimetry, which has almost the same outer geometry (diameter 6.9 mm). Therefore, the holder drilled for PTW‐31010 ionization chamber can potentially be used for the relative dosimetry measurements using the above four detectors if the effective point of measurement (EPM) of each detector can be correctly identified.

Four types of diode detectors dedicated to dose measurements in small beams were used in this study: PTW‐TN60008 Diode, PTW‐TN60016 Diode, PTW‐TN60017 Diode, and PTW‐60019 Diamond detectors. Figures 2(a)–2(d) are the pictures of these four diode detectors and its characteristics are listed in Table 1. As shown in Fig. 2, these four detectors have the same outer geometry (diameter 7 mm) but different internal structures. Figure 2(e) is the PTW‐31010 ionization chamber used for the reference dosimetry of the GKI at our institution. This ionization chamber has almost the same outer geometry with the diameter equal to 6.9 mm instead of 7 mm. Therefore, the holder customized for the PTW‐31010 ionization chamber can potentially be used for the relative dosimetry measurements using the above four diode detectors if the EPM of each detector can be correctly identified. As stated in the previous section, when the PTW‐31010 semiflex ion chamber is inserted into the sleeve of the holder of our Gamma Knife SW phantom, the center of the ion chamber or the EPM will be at Leksell coordinates of (100, 100, 100). However, (100,100,100) might not be the EPM for other detectors, particularly for diode detectors that have a much smaller sensitive volume than the PTW‐31010 ion chamber.

**Table 1:** Characteristics of the diode detectors used in this study. All four detectors have the same external but different internal geometry. The geometric form of sensitive area is “disk” for all detectors. The last column lists the z_eff_, which is the z‐Leksell coordinate in mm of effective measurement point (EMP) when it is inserted into the SW phantom. These values were derived in this study using the CBCT method.

Detector	Material	Sensitive volume (mm^3^)	Diameter or side length of sensitive volume (mm)	Thickness of sensitive volume (mm)	EMP from flat face/tip (mm)	z_eff_ (mm) identified from CBCT scans
PTW‐TN60008 diode	Silicon	0.03	1.13	0.03	2.0	98.5
PTW‐TN60016 diode	Silicon	0.03	1.13	0.03	2.4	98.9
PTW‐TN60017 diode	Silicon	0.03	1.13	0.03	1.3	97.8
PTW‐60019 diamond	Synthetic diamond	0.004	2.2	0.001	1.0	97.5

Since all four detectors have the same external geometry, the Leksell coordinates of the flat face/tips are the same. However, as illustrated in Table 1, the reference point measured from the flat face/tips is different for the four diode detectors. Given that the central axis of these four detector is along the z‐axis of Leksell coordinate system, the z coordinate is different for these four detectors while the x‐ and y‐ coordinates remain the same (100 and 100). It is therefore crucial to know the exact z‐ Leksell coordinate of EPM for each diode detector when inserted into the phantom; and position the phantom accordingly so that the EPM is located at UCP. Otherwise, the ROF measurements will not be accurate, particularly for the 4‐mm collimator.

### 2.D Determination of effective points of measurement

In this study, we developed a strategy for identifying the Leksell coordinates of EPM of a diode detector using the KV‐CBCT of GKI unit. For each diode detector, a CBCT image was acquired after it was inserted into the phantom and fixed with duct tape. Once the CBCT scan was localized, the flat face/tip of the detector was first identified in the images and the Leksell coordinates of EPM of that detector was determined based on the distance between the reference point and the flat face/tip provided in Table 1. Once the EPM of the diode was determined, a GKI plan was generated that aimed the radiation at the EPM coordinates instead of (100,100,100). When this new plan was executed, the couch would be moved to a location inside the GKI so that the Leksell coordinates of EPM, would coincide with the UCP. Sufficiently enough doses was prescribed for the plan so that the measurement time was set to one minute and were not controlled by the GKI plan but using the dose integration function (1 min integration) of the electrometer.

### 2.E Measurement of relative output along the z‐axis

In order to validate the identified EPM of a diode using the above‐mentioned CBCT method, a GKI plan was generated to measure the outputs of the 4‐mm collimator using PTW‐TN60017 diode at various couch locations along its central axis. Starting by aligning the UCP with Leksell coordinates (100, 100, 103), the couch was moved out in steps (0.1 or 0.2 mm for the in‐field and penumbra regions, and of 0.3 or 0.5 mm for the out‐of‐field region) along the z‐axis until the UCP coincided with Leksell coordinates (100, 100, 92). A large dose (e.g., 50 Gy) was prescribed for the plan in order to have enough irradiation time (on the order of an hour). In each stop, output was measured by integrating the ionization charge for a specified time larger than 30 s. The measured sensitivity for different locations along the z axis of diode was then plotted and the center of the irradiation field identified as the center to the two 50% profile levels was identified and compared with the EPM of PTW‐TN60017 identified using the CBCT method.

### 2.F Measurement of relative output factors

Output in term of ionization charge in nC (nanocoulombs) was measured for each collimator size using all four diode detectors and a PTW UNIDOS E electrometer. The exposure time (controlled by using the dose integration function of the electrometer) was one minute for each irradiation, and three readings were obtained and averaged for each collimator size. The measured output for each collimator size was used to obtain the ROF according to the recommendations of IAEA TRS483 report,^3^ particularly, Chapter 6 in that report for relative dosimetry of small fields. That is, the ROF was the ratio of the averaged readings for the 4‐mm/8‐mm collimator to that of 16‐mm collimator, multiplied by a field output correction factor, $k_{Q_{\mathrm{clin}},Q_{\mathrm{msr}}}^{f_{\mathrm{clin}},f_{\mathrm{msr}}}$ (given in table 25 of TRS483 for each detector) as a function of the collimator size, in order to correct for the side effects of small field sizes (Table 2). The same experiment was performed twice for each detector from which the average and standard deviation of the measured ROFs were calculated and compared with the vendor provided ROF in the treatment planning system, which were generated using Monte Carlo simulation.

**Table 2:** Field output correction factors,$k_{Q_{\mathrm{clin}},Q_{\mathrm{msr}}}^{f_{\mathrm{clin}},f_{\mathrm{msr}}}$, in IAEA TRS‐483 Code of Practice for the Gamma Knife, as a function of the diameter of the circular collimator.

Detectors	Output correction factor,$k_{Q_{clin},Q_{msr}}^{f_{clin},f_{msr}}$		
	4 mm	8 mm	16 mm
PTWTN60008 diode	0.951	0.971	1.000
PTW‐TN60016 diode	0.958	0.981	1.000
PTW‐TN60017 diode	0.961	0.997	1.000
PTW‐60019 diamond	0.993	1.005	1.000

## 3 RESULTS

**Fig. 3:**
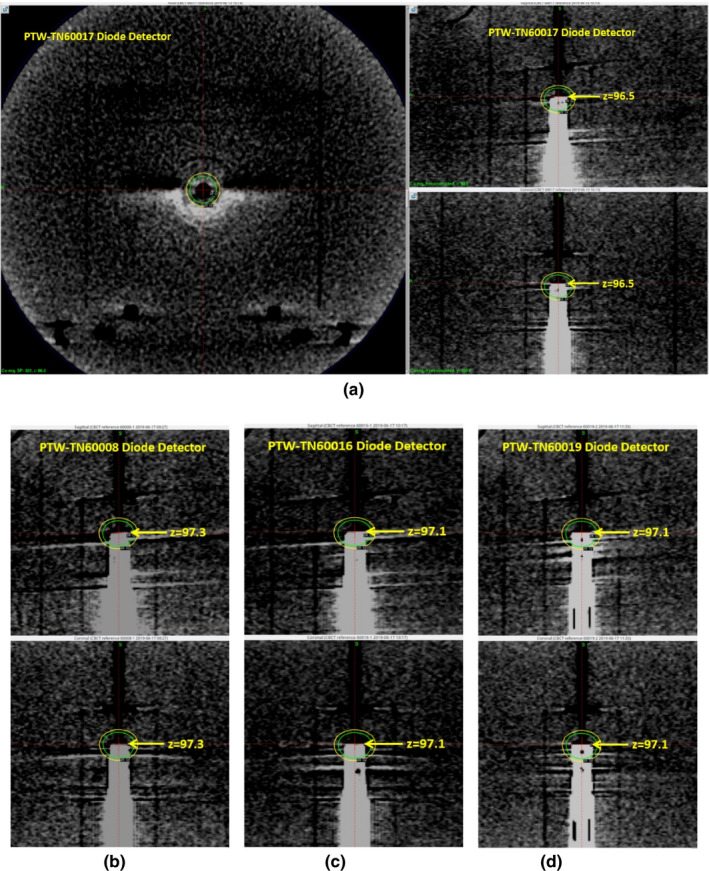
Axial (left), sagittal (right upper), and coronal (right left) views of (a) the CBCT scan of the PTW‐TN60017 diode detector. The yellow line in each panel marks the most superior part of the detector that can be identified from the scan. Also shown in (b)–(d) are the sagittal (upper) and coronal (lower) views of the other three diode detector.

Figure 3(a) shows the axial, sagittal, and coronal views of (a) the CBCT scan of the PTW‐TN60017 diode detector. The yellow line in each panel marks the most superior part of the detector that can be identified from the scan. Figures 3(b)–3(d) are the sagittal and coronal views of the other three detectors. Given that all four detectors have the same external geometry, the smallest z‐ Leksell coordinate (z = 96.5 mm identified from the scan of PTW‐TN60017 diode detector) was chosen as the z‐ Leksell coordinate of the flat face/tip for all four detectors.

**Fig. 4:**
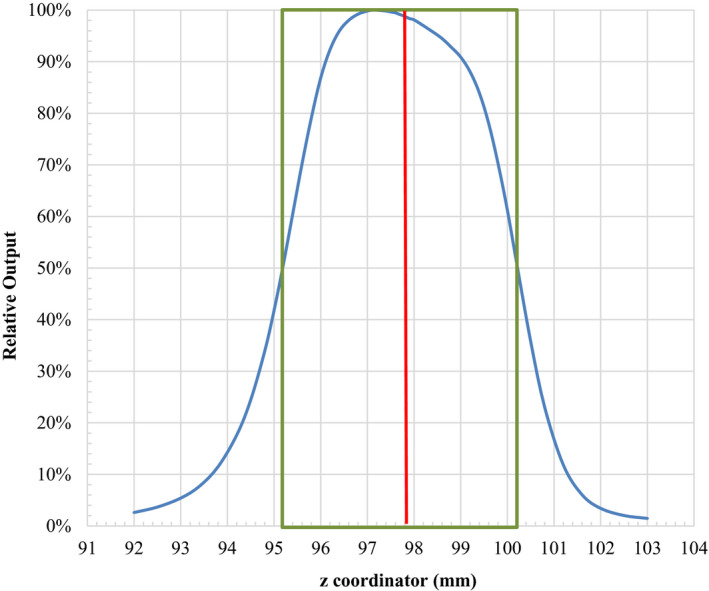
Relative output of the 4‐mm shot at different location along its z‐axis, measured using the PTW‐TN60017 diode detector. The 50% relative output (green box) occurs at z = 95.2 mm and 100.2 mm, so the center of the measured profile is located at (95.2 + 100.2)/2 = 97.7 mm, which is 0.1 mm different from the identified location (z_eff_ = 97.8 mm, red line) of the sensitive volume for this diode.

This chosen value was validated by measuring the relative output of the 4‐mm shot along the z‐axis using the PTW‐TN60017 diode detector. Figure 4 plots the measured relative output at different couch location along its z axis. The 50% relative output (greed box) occurs at z = 95.2 mm and 100.2 mm, so the full width half maxima (FWHM) of the beam is 100.2 − 95.2 = 5.0 mm, which is 0.01 mm larger than the FWHM (4.99 mm) obtained from the machine commissioning. The center of the measured profile is located at (95.2 + 100.2)/2 = 97.7 mm, which is 0.1 mm different from the location (z_eff_ = 97.8 mm, red line) of the sensitive volume for this diode identified using the CBCT method.

**Fig. 5:**
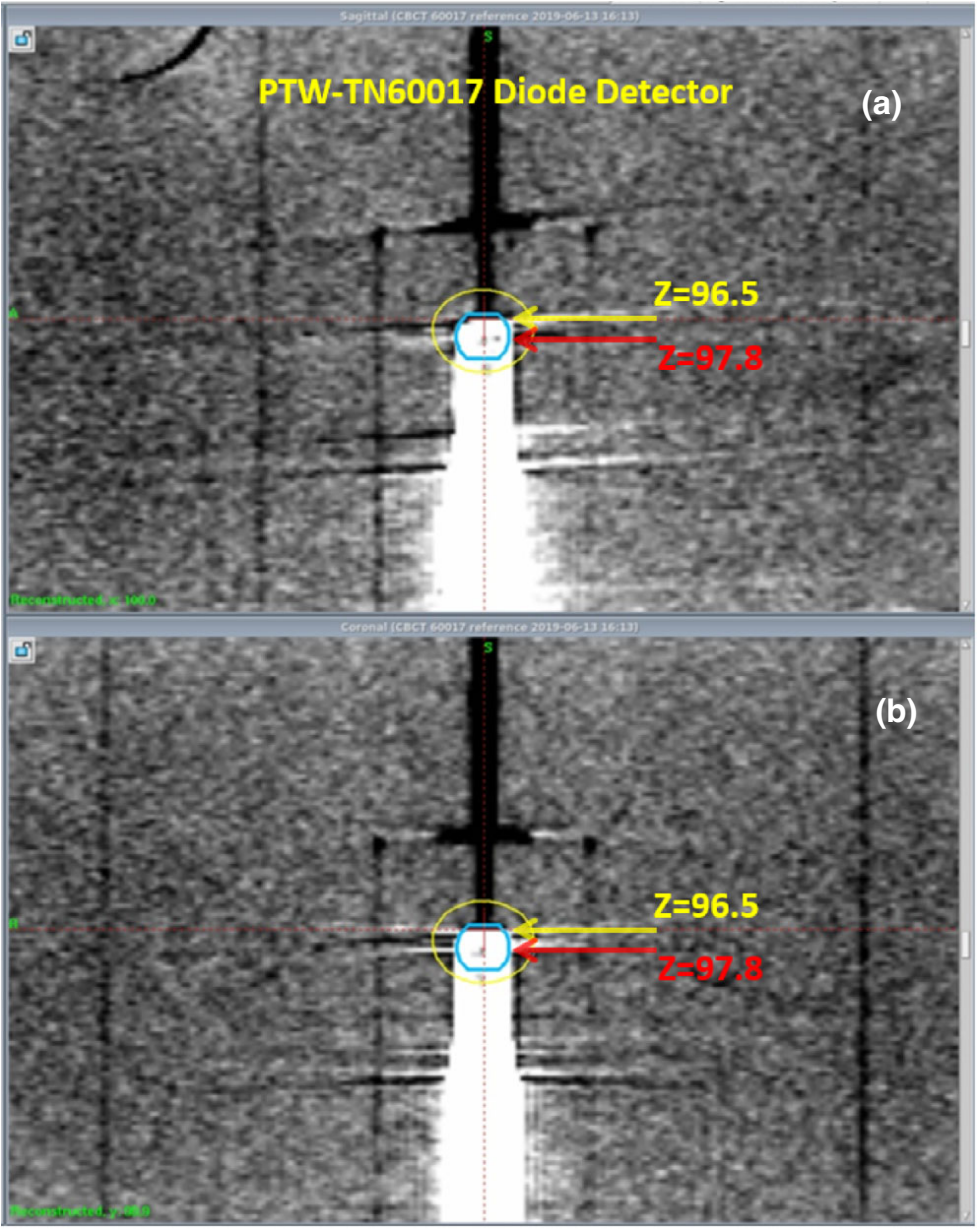
Illustration of the identification of effective point of measurement (EPM) using the CBCT images of the PTW‐60017 diode detector: (a) sagittal and (b) coronal view. The yellow lines mark the Leksell coordinator z = 96.5 (mm) of the tip of detector in the z‐ or superior‐inferior direction. The red lines indicate the location of EPM. Since the EPM is 1.3 mm inferior from the tip for this diode (Table 1), z_eff_ = 96.5 + 1.3 = 97.8 (mm) is the z‐ Leksell coordinate of the sensitive volume in the z direction.

Figure 5 illustrates the identification of EPM for the PTW‐60017 diode detector. The z‐ Leksell coordinate of the flat face/tip of that detector was at z = 96.5 (note that both x‐ and y‐ Leksell coordinates remain at 100 and are not shown). From Table 1, the EPM for the PTW‐60017 diode detector is at 1.3 mm from the tip. Thus, the exact location of sensitive volume is at z_eff_ = 96.5 + 1.3 = 97.8 (mm), which is 2.2 mm from z = 100 mm. The same calculation was performed for all four diodes used in this study and the results were tabulated in the last column of Table 1.

The OFs measured as a function of collimator size for all four diode detectors uncorrected for the location of sensitive volume (i.e., assuming the sensitive volume is located at z_eff_ = 100 mm, or the geometric center of the phantom), the ROFs in the GKP system calculated from Monte Carlo^12^ and their percent differences are listed in the Tables 3 and 4. As shown in Table 4, significant differences were observed between the measured and reference if the EPM of the diode is not positioned at machine UCP.

**Table 3:** Relative Output factors (ROFs, relative to the 16 mm collimator) for the Gamma Knife Icon uncorrected for the location of sensitive volume, that is, assuming the sensitive volume is located at z‐Leksell coordinate z_eff_ = 100 mm, or the geometric center of the phantom. All measurements were performed with the same plan. The reference ROFs are that used in Gamma Knife planning (GKP) system, which were calculated using Monte Carlo simulations.

Shot size	Uncorrected ROFs		
	4 mm	8 mm	16 mm
PTW‐TN60008 diode	0.812	0.894	1.00
PTW‐TN60016 diode	0.792	0.900	1.00
PTW‐TN60017 diode	0.478	0.863	1.00
PTW‐60019 diamond	0.623	0.867	1.00
Reference ROFs	0.814	0.9005	1.00

**Table 4:** Percent difference between the uncorrected ROFs measured with diode detectors and that used in GKP.

Collimator Size (mm)	PTW‐TN60008 diode (%)	PTW‐TN60016 diode (%)	PTW‐TN60017 diode (%)	PTW‐60019 diamond (%)
4	0.3	2.7	41.3	23.5
8	0.7	0.1	4.2	3.7

**Fig. 6:**
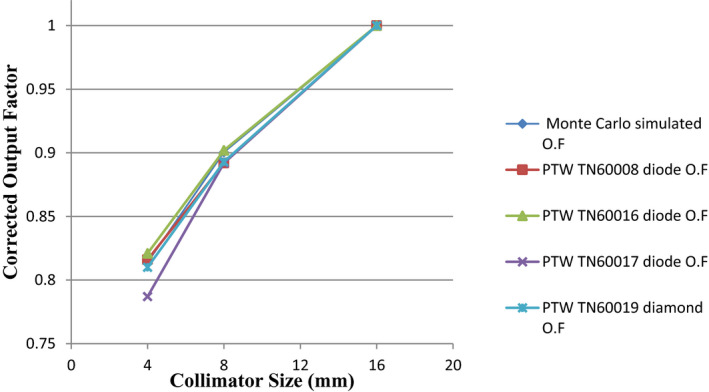
Relative out factors (ROFs) measured with different diodes on a Leksell Gamma Knife® Icon™. ROF is equal to 1 for the 16‐mm collimator size as all measured outputs were normalized to that of the 16‐mm collimator.

Table 5 lists the mean and standard deviation of the ROFs of 4 and 8‐mm collimators for the GKI relative to the 16‐mm collimator, measured at the EPM of each detector determined using the CBCT method. The same data are plotted in Figure 6 to demonstrate the good agreement between the measured and reference ROFs. Table 6 tabulates the percent difference (mean ± standard deviation) between the corrected ROFs measured with diode detectors and that used in GKP. For PTW‐TN60008 diode, the percentage variations were 0.6 ± 0.4%, and −0.8 ± 0.2% for 4 and 8 mm collimators, respectively. For PTW‐TN60016 diode, the percentage variations were 0.8 ± 0.0%, and 0.2 ± 0.1%, respectively. The percentage variations were −3.3 ± 0.0% and −0.9 ± 0.1%, respectively, for PTW‐TN60017 diode, and −0.5 ± 0.0% and −0.8 ± 0.2%, respectively, for the PTW−60019 diamond detector (Table 6).

**Table 5:** Average and standard deviation of the relative output factors (ROF, relative to the 16 mm collimator) for the Gamma Knife corrected for the location of sensitive volume of each detector. A customized plan was generated for each detector. For each experiment, three readings were taken for each collimator size, and its average were used to calculate the ROF. The same experiment was performed twice for each detector from which the average and standard deviation of the measured ROFs were calculated and tabulated in this table. The reference ROFs are that used in Gamma Knife planning (GKP) system, which were calculated using Monte Carlo simulations.

Shot size	Corrected ROFs		
	4 mm	8 mm	16 mm
PTW‐TN60008 diode	0.819 ± 0.003	0.893 ± 0.002	1.00
PTW‐TN60016 diode	0.821 ± 0.000	0.903 ± 0.001	1.00
PTW‐TN60017 diode	0.787 ± 0.000	0.893 ± 0.000	1.00
PTW‐60019 diamond	0.810 ± 0.000	0.894 ± 0.001	1.00
Reference ROFs	0.814	0.9005	1.00

**Table 6:** Percentage difference (average ± standard deviation) between the corrected ROFs measured with diode detectors and that used in GKP.

Collimator size (mm)	PTW‐TN60008 diode (%)	PTW‐TN60016 diode (%)	PTW‐TN60017 diode (%)	PTW‐60019 diamond (%)
4	0.6 ± 0.4	0.8 ± 0.0	−3.3 ± 0.0	−0.5 ± 0.0
8	−0.8 ± 0.2	0.2 ± 0.1	−0.9 ± 0.1	−0.8 ± 0.2

## 4 DISCUSSION

In this study, we developed a novel and simple method to validate the relative collimator OFs for Leksell Gamma Knife® Icon™ using diode detectors and existing SW phantom. The SW phantom at our Center was configured for PTW‐31010 ion chamber, that is, the EPM of the ion chamber is at Leksell coordinates (100,100,100) when inserted into the SW phantom. Since the diode detectors have different geometric configurations, their EPMs will have different Leksell coordinates when inserted into the phantom. Therefore, we scanned each detector using the GKI CBCT to obtain the correct Leksell coordinates for each diode and reprogrammed the treatment plan for output measurements so that the diode’s EPM and machine UCP were aligned properly.

Even though the image quality of the CBCT scans in Fig. 2 is not optimal, it is observed in this figure that the internal structure is very different for these four detectors. This is particularly obvious when we compare the z‐Leksell coordinates of the most superior part of the detector that can be identified from the scans of these four detectors: they are quite different ranging from 96.5 to 97.3 mm. This large variation was attributed to the different internal structures, particularly the presence of air gaps in some of these four detectors. Therefore, even though these four detectors have the same external geometry (outer diameter 7 mm), the tip/front wall might not show in the CBCT scan if it is next to low attenuation material (e.g., air gaps).

Because the CBCT image quality was not optimal, we decided to perform an independent check by measuring the relative output of the 4‐mm collimator using the PTW 60017 detector. The FWHM of the measured profile agreed very well with that determined during the commissioning and the z Leksell coordinate of the center measured profile was only 0.1 mm different from that determined using the CBCT method. It is noticed that the shape of the profile in Fig. 4 is not symmetrical around the center of the field but is slightly higher (~2%) toward the superior side. This was most likely because the beam was attenuated slightly more when the detector traveled toward the inferior side. Given the fast falloff of beam intensity in the penumbra region (80%–20% in 1.4 mm), this slight variation in beam intensity would have caused a minor (<0.1 mm) difference in the measured FWHM and the location of beam center. Therefore, we were confident that z = 96.5 mm is the z Leksell coordinate of the tip/front wall.

The ROF measurements were performed following the recommendations of IAEA TRS‐483 report^3^ for relative dosimetry of small fields. In general, the differences in the measured ROFs were within 1% of the recommended OFs used by the treatment planning system, as shown in Tables 5 and 6 and Fig. 3. The only exception was the PTW‐TN60017 diode which has underestimated the ROF by 3.3% for the 4‐mm collimator. Even though the 3.3% accuracy is generally acceptable for validating GK collimator ROFs, particularly for the 4‐mm collimator, we still repeated multiple CBCT images and measurements for this particular diode. The results in Fig. 4 showed that the EPM identified using the CBCT scan was correct but the deviations in the output factor were similar after multiple measurements. Therefore, we concluded that the deviations could be due to the fact that the $k_{Q_{\mathrm{clin}},Q_{\mathrm{msr}}}^{f_{\mathrm{clin}},f_{\mathrm{msr}}}$for PTW‐TN60017 diode might not be sufficient enough to correct for the field output at 4mm collimator size.

Hrsak H et al. have determined a correction factor for the angular dependence of PTW‐TN60016 and PTW‐TN60017 and the final Gamma Knife ROFs were corrected for this dependence.^9^ In the case of 8mm collimator, both diodes showed ±0.6% agreement with the recommended values. Meanwhile, for the 4 mm collimator, both diodes showed an over response even after applying all the correction factors. However, in our study, for the PTW‐TN60016 diode, the result for the 4 and 8 mm collimations are better and is in good agreement with the Monte Carlo calculated values even without the correction for the angular dependence.

However several authors reported silicon detectors will over respond to lower energy scattered photons due to photo electric effect in the sensitive area of the silicon.^13^ To reduce this effect in unshielded PTW‐TN60017, a disk‐shaped silicon chip surrounded by a polymer plastic is fixed to reduce the unwanted back scattering of electrons from the shield.^14^ Hence the discrepancies could be possibly due to the increase of backscattered electrons from this inefficient shield as the angle of incident photon beam decreases. Further study is needed to identify the source of this discrepancy.

Alternate detectors such as polymer gels, radiographic film and radiochromic film can also be considered for the validation of ROF as there are no ideal detectors for the ROF measurement of the Gamma Knife Unit. Moutsatsos et al.,^15^ have used water equivalent polymer gel dosimeters for the ROF measurements of Gamma Knife model C unit for the 4‐ and 8‐mm collimators relative to the 18‐mm collimator. The ROFs for the 8‐mm collimator were in close agreement with vendor recommended value. However for the 4‐mm collimator, the ROFs were 3% lower than the value recommended by the vendor.

A study by Ma et al.^5^ has used a single EBT film for the ROF measurement of a Leksell Gamma Knife Perfexion unit. They used a double‐shot exposures method to validate the same and the measured ROFs were <2% for both the 4‐ and 8‐mm collimators. Similar results were obtained for Novotny et al.^8^ with EBT Gafchromic films and the results were <1.5% for the 4 and 8‐mm collimators using a sequential exposure method. However, for the Kodak EDR 2 films, the percentage differences were −2.1% and −4.5% for 8‐ and 4‐mm collimators, respectively.

A recent study by Michele Zeverino et al.^16^ has validated the collimator output factor for Leksell Gamma Knife Icon using EBT3 films. The ROFs for the 8 and 4 mm collimators were found to be 0.887 ± 0.035 and 0.797 ± 0.032, respectively. The results were within 2% of the Monte Carlo‐derived values and are comparable with our data. Thus radiochromic films can be an interesting alternative to diode detectors because of their excellent spatial resolution which solves the issue related to partial volume irradiation. However, they also have encountered several setbacks which were mostly related to the calibration process and could introduce larger uncertainties.

## 5 CONCLUSION

In this study, a novel and simple imaging method was developed to determine the EPMs of diode detectors when inserted into the existing LGK‐SW phantom. With the acquired information, each diode could be positioned at the focal point of the machine for relative collimator OF measurements, without any modification or customization of the phantom. Following the recommendations of IAEA TRS‐483 report^3^ for relative dosimetry of small fields, the ROFs measured using this method showed a very good agreement with the values recommended by the manufacturer for all diode detectors used in this study. The only exception was the measurement of ROF for the 4‐mm collimator using the PTW‐TN60017 diode detector. We suspect it was due to some unknown discrepancy of the output correction factor, $k_{Q_{\mathrm{clin}},Q_{\mathrm{msr}}}^{f_{\mathrm{clin}},f_{\mathrm{msr}}}$for that diode, and further investigation is needed.

## CONFLICT OF INTEREST

The authors declare no conflict of interest.
